# Integrative Multi-Kinase Approach for the Identification of Potent Antiplasmodial Hits

**DOI:** 10.3389/fchem.2019.00773

**Published:** 2019-11-21

**Authors:** Marilia N. N. Lima, Gustavo C. Cassiano, Kaira C. P. Tomaz, Arthur C. Silva, Bruna K. P. Sousa, Leticia T. Ferreira, Tatyana A. Tavella, Juliana Calit, Daniel Y. Bargieri, Bruno J. Neves, Fabio T. M. Costa, Carolina Horta Andrade

**Affiliations:** ^1^LabMol—Laboratory for Molecular Modeling and Drug Design, Faculty of Pharmacy, Federal University of Goiás, Goiânia, Brazil; ^2^Laboratory of Tropical Diseases—Prof. Dr. Luiz Jacintho da Silva, Department of Genetics, Evolution, Microbiology and Immunology, Institute of Biology, University of Campinas (UNICAMP), Campinas, Brazil; ^3^Department of Parasitology, Institute of Biomedical Sciences, University of São Paulo, São Paulo, Brazil; ^4^Laboratory of Cheminformatics, University Center of Anápolis/UniEVANGELICA, Anápolis, Brazil

**Keywords:** malaria, shape-based, machine learning, virtual screening, *Plasmodium falciparum*, multi-target

## Abstract

Malaria is a tropical infectious disease that affects over 219 million people worldwide. Due to the constant emergence of parasitic resistance to the current antimalarial drugs, the discovery of new antimalarial drugs is a global health priority. Multi-target drug discovery is a promising and innovative strategy for drug discovery and it is currently regarded as one of the best strategies to face drug resistance. Aiming to identify new multi-target antimalarial drug candidates, we developed an integrative computational approach to select multi-kinase inhibitors for *Plasmodium falciparum* calcium-dependent protein kinases 1 and 4 (CDPK1 and CDPK4) and protein kinase 6 (PK6). For this purpose, we developed and validated shape-based and machine learning models to prioritize compounds for experimental evaluation. Then, we applied the best models for virtual screening of a large commercial database of drug-like molecules. Ten computational hits were experimentally evaluated against asexual blood stages of both sensitive and multi-drug resistant *P. falciparum* strains. Among them, LabMol-171, LabMol-172, and LabMol-181 showed potent antiplasmodial activity at nanomolar concentrations (EC_50_ ≤ 700 nM) and selectivity indices >15 folds. In addition, LabMol-171 and LabMol-181 showed good *in vitro* inhibition of *P. berghei* ookinete formation and therefore represent promising transmission-blocking scaffolds. Finally, docking studies with protein kinases CDPK1, CDPK4, and PK6 showed structural insights for further hit-to-lead optimization studies.

## Introduction

Malaria is a serious infectious disease that affects 219 million people worldwide and kills over 435,000 patients annually, especially pregnant women and children in Sub-Saharan Africa (WHO, 2018). The disease is transmitted to humans through the bites of infected female *Anopheles* mosquitoes and caused by *Plasmodium* genus parasites (Ashley et al., 2018). Among them, *P. falciparum* is the most devastating species responsible for severe form of malaria and deaths (WHO, 2017).

Current control and eradication demands a combination of drugs with different mechanisms of action. Despite of compelling investment for controlling and eliminating this infectious disease, resistant parasite strains have been reported to all major antimalarial drugs (Wu et al., 1996; Triglia et al., 1998; Srivastava et al., 1999; Wellems and Plowe, 2001), including front-line artemisinin-based combination therapies (Rogers et al., 2009; Witkowski et al., 2013; Ashley et al., 2014). All these aspects highlight the urgent need for the discovery of new antimalarial drugs by identifying molecules with novel mechanisms of action and efficient against resistant parasite strains (Burrows et al., 2017).

The complete genome sequencing of *P. falciparum* (Gardner et al., 2002) has provided new and valuable information on its biological pathways, identifying potentially relevant biological targets for therapeutic intervention. In this context, protein kinases have been investigated because of their importance in several essential signaling pathways, e.g., homeostasis, apoptosis and cell division (Lucet et al., 2012; Bullard et al., 2013). Kinases catalyze the transfer of phosphate groups from ATP to specific substrates. These enzymes share a high degree of sequence and structural homology between the ATP binding sites, making them potential targets to be grouped and inhibited simultaneously by a single molecule. This mechanism, known as multi-kinase inhibition (MKI), provides a synergistic effect responsible for increasing the effectiveness of the kinase inhibitors, and consequently preventing the emergence of parasite resistance (Garuti et al., 2015). On the other hand, promiscuity is the main challenge in parasitic MKI design, which requires selective inhibitors unable to interact with host protein (Davies et al., 2000; Bain et al., 2003, 2007). However, the vast phylogenetic distance between Apicomplexans and humans (Ward et al., 2004) makes possible the development of multi-target and selective antimalarial candidates.

Calcium-Dependent Protein Kinases (CDPKs), a kinase family of plants and some alveolates, absent in metazoans, have been considered as one of the main effectors of calcium signaling, demonstrating a pronounced importance in apicomplexans, controlling a range of events in the parasite life cycle (Nagamune et al., 2008). *Pf* CDPK1 is expressed in all *Plasmodium* life stages (Sebastian et al., 2012), being essential for the sexual stage of the parasite (Jebiwott et al., 2013; Bansal et al., 2018). Meanwhile, *Pf* CDPK4 regulates cell cycle progression in the male gametocyte (Billker et al., 2004) and, together with Protein Kinase G, is activated during hepatocytes invasion by sporozoite (Govindasamy et al., 2016). Protein Kinase 6 of *P. falciparum* (*Pf* PK6), classified as Cyclin-Dependent Kinase (Chakrabarti et al., 1993), appears to be located in the cytoplasm and nucleus, mainly expressed in trophozoite, schizonts and segmenters stages (Bracchi-Ricard et al., 2000). The low identity between *Pf* PK6 and human Cyclin-Dependent Kinase 2 brings out PK6 as a potential antimalarial target. Its numerous variations in the active site amino acids can be exploited to design selective plasmodial inhibitors (Waters and Geyer, 2003). Therefore, the structural dissimilarities between human kinases and *Plasmodium*-specific kinases, such as CDPK1 and CDPK4 and PK6, turn these enzymes attractive targets for development of new multi-target antimalarial therapies (Lucet et al., 2012; Crowther et al., 2016). Recently, Crowther and colleagues (Crowther et al., 2016) reported an experimental screening of ~14,000 cell-active compounds against *Pf* CDPK1 and *Pf* CDPK4, mitogen-associated protein kinase 2, PK6, and protein kinase 7. They found potent inhibitors (IC_50_ <1 μM) for multiple kinases simultaneously, with low cytotoxicity to human, bypassing the challenging of MKI promiscuity. Thus, the availability of the whole dataset of compounds with data for kinase inhibition allowed us to generate and validate robust and predictive shape-based models, that were integrated with machine learning (ML) models for a virtual screening workflow aiming to prioritize compounds to be experimentally evaluated *in vitro* against asexual blood stages of both sensitive and multi-drug resistant *P. falciparum*, and against sexual stages of *P. berghei*, as well as in mammalian cells. This integrative analysis allowed us to identify new potential and selective antiplasmodial hits.

## Materials and Methods

**Figure 1:**
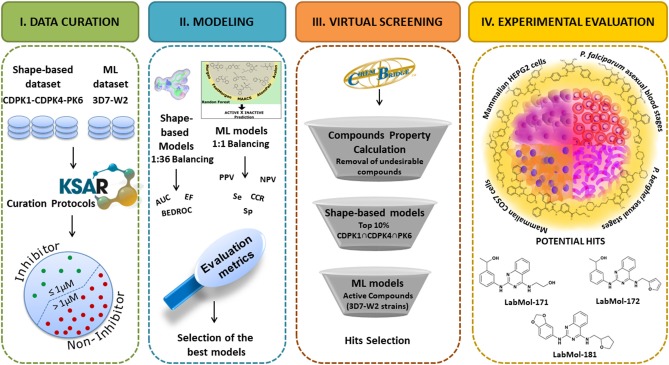
Overall pipeline designed for the identification of new antiplasmodial compounds.

The overall study design is shown in Figure 1. Briefly, we followed the successive steps: (I) dataset collection, curation, and integration of compounds with activity against CDPK1, CDPK4, PK6, and asexual-blood stages of *P. falciparum*; (II) development of shape-based models for CDPK1, CDPK4, and PK6, and machine learning models for *P. falciparum*; (III) virtual screening of ChemBridge database (~1 million compounds); and (IV) experimental validation of prioritized compounds against asexual blood stage of *P. falciparum* (sensitive and multi-drug resistant strains), sexual blood stages of *P. berghei* and cytotoxicity in mammalian cells.

### Computational

The whole project was built envisioning best practices of ML modeling (Tropsha, 2010; Cherkasov et al., 2014).

#### Data Integration and Curation

In this study, five datasets extracted from the PubChem Bioassay database (Wang et al., 2012) were explored to build shape-based models and ML models. All datasets were carefully standardized according to the protocol described by Fourches et al. (2010, 2015, 2016). Thus, explicit hydrogens were added; counter ions, inorganic salts, polymers, mixtures, and organometallic compounds were removed; and specific chemotypes (aromatic, nitro groups and others) were normalized using ChemAxon Standardizer (v. 6.1, ChemAxon, Budapest, Hungary, www.chemaxon.com). Then, a go/no-go criteria of 1 μM for the progression of *P. falciparum* kinase inhibitors and antiplasmodial hits (Katsuno et al., 2015) was used as activity threshold to distinguish active vs. inactive compounds. Furthermore, we performed the analysis and exclusion of duplicates as follows: (a) if the reported outcomes of the duplicates were the same, one entry was retained in the dataset and the other excluded, and (b) if duplicates presented discordance in biological activity, both entries were excluded from dataset. A brief description of the datasets is presented below.

- CDPK1: 181 active compounds with IC_50_ ≤ 1 μM and 13,270 inactive compounds with IC_50_ >1 μM (National Center for Biotechnology Information, 2016a);
- CDPK4: 55 active compounds with IC_50_ ≤ 1 μM and 13,396 inactive compounds with IC_50_ >1 μM (National Center for Biotechnology Information, 2016b);
- PK6: 65 active compounds with IC_50_ ≤ 1 μM and 13,386 inactive compounds with IC_50_ >1 μM (National Center for Biotechnology Information, 2016c);
- *P. falciparum* 3D7 (drug-susceptible) strain: 3,497 active compounds with EC_50_ ≤ 1 μM and 4,376 inactive compounds with EC_50_ >1 μM (National Center for Biotechnology Information, 2009a, 2010a, 2011a, 2013);
- *P. falciparum* W2 (drug-resistant) strain: 3,637 active compounds with EC_50_ ≤ 1 μM and 3,766 inactive compounds with EC_50_ >1 μM (National Center for Biotechnology Information, 2009b, 2010b, 2011b, 2012).

The inhibitory activity against each kinase was considered proportional to ATP consumed, as determined from measurements of residual [ATP] with the luciferase-based assay. So, all active compounds used to build shape-based models are inhibitors of ATP binding site (Crowther et al., 2016). All datasets generated for this study are included in the manuscript and the Supplementary Files.

#### Shape-Based Models

The shape-based models were built to distinguish active vs. inactive compounds for *P. falciparum* CDPK1, CDPK4, and PK6. Initially, the curated datasets were balanced by linear under-sampling method obeying a proportion of 1:36, aiming to reproduce the chemical space of an HTS, which contain more non-inhibitors. Then, 200 conformations were generated for each compound using OMEGA v.2.5.1.4 software (OMEGA 2.5.1.4: OpenEye Scientific Software, Santa Fe, NM. http://www.eyesopen.com) (Hawkins et al., 2010), while the protonation states at neutral pH and AM1-BCC charges (Jakalian et al., 2002) were estimated using QUACPAC v.1.7.0.2 (QUACPAC 1.7.0.2: OpenEye Scientific Software, Santa Fe, NM. http://www.eyesopen.com). To create the shape-based models, the most potent compounds against each kinase (see details in Table S1) were loaded into ROCS software v.3.2.2.2 (ROCS 3.2.2.2: OpenEye Scientific Software, Santa Fe, NM. http://www.eyesopen.com) (Hawkins et al., 2007) and used as query compound. Then, the output conformations of active and inactive compounds were aligned by a solid-body optimization process that maximizes the overlap volume with queries, and ranked according to *Reference Tversky Combo* scoring function (Hawkins et al., 2007). Finally, the predictive performance of the shape-based models was assessed using the following metrics: Receiver Operating Characteristic (ROC) curve, Area Under the ROC Curve (AUC), Boltzmann-Enhanced Discrimination of ROC (BEDROC) and Enrichment Factor (EF). These statistic metrics are calculated by the following equations:

(1) $$\begin{array}{r} AUC=\;\underset{i}{\sum }\left[\left(SE_{i+1}\right)\left(SP_{i+1}-\;SP_{i}\;\right)\right] \end{array}$$

(2) $$\begin{array}{l} BEDROC=RIE\;\times \;\frac{R_{a}sinh\left(\frac{\alpha }{2}\right)}{cosh\left(\frac{\alpha }{2}\right)-cosh\left(\frac{\alpha }{2}-\;\alpha R_{a}\right)}\\ \;+\;\frac{1}{1-e^{\alpha \left(1-R_{a}\right)}}\;\approx \;\frac{RIE}{\alpha }\\ \;+\;\frac{1}{1-e^{\alpha }},if\;\alpha R_{a}\;\ll 1\;and\;\alpha \neq 0 \end{array}$$

(3) $$\begin{array}{r} EF^{x\%}=\;\frac{Hits_{selected}^{x\%}/N_{selected}^{x\%}}{Hits_{total}/N_{total}} \end{array}$$

Here, *SE* denotes sensitivity and *SP* specificity, RIE robust initial enhancement, *R*_*a*_ ratio of actives in the list.

The ROC curve provides a graphical representation of a predictor's behavior by plotting the true (Braga and Andrade, 2013; Neves et al., 2016) positive rate [sensitivity (SE)] against the 1 minus false positive rate [1—Specificity (SP)]. See SE e SP equations in ML section. The ideal predictive model would yield a point in the upper left corner of the ROC plot, representing 100% *SE* and *SP*. The AUC is the probability that a model will rank an active compound higher than a randomly chosen inactive. The EF shows how many times the shape-based models retrieved active compounds when compared with random selection (Braga and Andrade, 2013). Lastly, BEDROC uses an exponential decay function to favor models that pile up active compounds near the top of the rank-ordered list from the virtual screening (Huang and Wong, 2016).

#### Machine Learning Models

Binary ML models were built to distinguish active vs. inactive compounds for *P. falciparum*. The curated datasets for *P. falciparum* 3D7 and W2 strains were balanced in a proportion of 1 active:1 inactive. For this, the original chemical space of each library was maintained through linear under-sampling method based on k-nearest neighbors distances of each inactive to all active. ML models were built using an in-house workflow, implemented in KNIME (Berthold et al., 2009) including many modules as multiple machine learning methods, performance metrics, applicability domain, and Y-randomization test. Five molecular fingerprints implemented in RDKit (v.2.4.0) (http://www.rdkit.org) were used: (i) Morgan and (ii) FeatMorgan fingerprints, generated using radius of 2 and bit vector of 1,024 bits (Morgan, 1965; Rogers and Hahn, 2010); (iii) Molecular ACCess System (MACCS) structural keys (Dill et al., 1981; Anderson, 1984; Durant et al., 2002); (iv) AtomPair fingerprint with bit vector of 1,024 bits and path length ranging between 1 and 10 (Carhart et al., 1985); and (v) Avalon fingerprint with bit vector of 1,024 bits (Gedeck et al., 2006). The Random Forest method was the chosen algorithm to generate the models and to produce the final prediction based on combination of each decision tree (Breiman, 2001; Svetnik et al., 2003).

Moreover, for ML models' robustness estimation, 5-fold external cross-validation was performed. In this method, each dataset is randomly and equally divided into five subsets. Then, one of them is outwardly maintained as external set and the remaining four establish the modeling set. This procedure is repeated five times, allowing each subset to be used once as external validation set. The performance and robustness of ML models were assessed through statistic metrics such as: sensitivity (SE), specificity (SP), Correct Classification Rate (CCR), Positive Predictive Value (PPV), and Negative Predictive Value (NPV). These statistic metrics are calculated by the following equations:

(4) $$\begin{array}{r} SE=\frac{TP}{TP+FN} \end{array}$$

(5) $$\begin{array}{r} SP=\frac{TN}{TN+FP} \end{array}$$

(6) $$\begin{array}{r} CCR=\frac{SE+SP}{2} \end{array}$$

(7) $$\begin{array}{r} PPV=\;\frac{TP}{TP+FP} \end{array}$$

(8) $$\begin{array}{r} NPV=\;\frac{TN}{TN+FN} \end{array}$$

Here, TP and TN correspond respectively to the number of true positives and true negatives. FP and FN represent, respectively, the number of false positives and false negatives.

In addition, 10 rounds of Y-randomization were conducted to evaluate whether the correlation between structure and activity occurred by chance. To measure the reliability of developed ML models, the Applicability Domain (AD) was estimated using Euclidean distances between each external compound, obtained by 5-fold cross-validation procedure, and their respective nearest neighbor in modeling set. These distances were related to the pre-defined AD threshold level. Toward a pre-defined distance threshold, the distance superior to this threshold were considered unreliable (Zhang et al., 2006).

In this study, we defined AD as:

(9) $$\begin{array}{r} D_{T}=\;\bar{y}+Z\sigma \end{array}$$

Here, DT is a distance threshold, $\bar{y}\;$is the average Euclidean distance of the k nearest neighbors of each compound of the training set, σ represents the standard deviation of the Euclidean distances and *Z* is an arbitrary parameter to control the level of significance. We set the default value of 0.5 for *Z*.

Consensus modeling was done combining the best ML models of each fingerprint type with Random Forest machine learning method. This approach was adopted with the aim to capture the different chemical information provided by each fingerprint, enriching the prediction during virtual screening and minimize individual model's error. Each individual model was applied to predict the activity of selected compounds after passing through shape-based screening filter. For this purpose, five models for 3D7 strain and five models for W2 strain were employed in separate runs. This way, when a model predicted a compound as active, a value of 0.2 was given, thus the final value of probability to be active was ranging from 0 to 1. Only compounds inside AD and predicted as active at least in three models (probability ≥ 0.6) of both strains were picked up.

#### Virtual Screening

Developed shape-based and ML models were used for VS of ~1.1 million compounds available on ChemBridge database (http://www.chembridge.com/) aiming to identify new potential kinases inhibitors with antiplasmodial activity. Prior to screening, the database was filtered using Veber (Veber et al., 2002) and Lipinski's rules (Lipinski et al., 2001) to prioritize drug-like molecules, using FILTER (OMEGA 2.5.1.4: OpenEye Scientific Software, Santa Fe, NM. http://www.eyesopen.com). Subsequently, molecules were filtered by shape-based models developed for CDPK1, CDPK4, and PK6. Then, the common compounds between the top 10% of each kinase list had their antiplasmodial activity predicted by consensus ML models developed for 3D7 and W2 strains. The compounds prediction were recognized if it were found within the AD of more than 50% of all models used in consensus prediction. Finally, the selected virtual hits were purchased and submitted to *in vitro* experimental evaluation.

#### Homology Modeling

The amino acid sequence of *P. falciparum* CDPK1 and PK6 were not available on the Protein Data Bank at the time this work was conducted. Consequently, homology models were built by comparing the *P. falciparum* primary sequences with sequences of homolog proteins (templates) whose 3D structures were publicly available. Initially, the sequences of *P. falciparum* kinases were extracted from the UniProt database (Apweiler, 2004) and used as target for homology modeling in the SWISS-MODEL webserver (Bordoli et al., 2009; Biasini et al., 2014). Then, the tailored models were structurally optimized in GalaxyWEB server (Ko et al., 2012). Finally, overall stereochemical and geometrical quality of refined models were investigated using MolProbity server (Chen et al., 2010).

#### Docking

Chemical structures of antiplasmodial hits were imported to Maestro v. 10.7.015 (Schrödinger, LLC, New York, NY, 2016) and prepared using LigPrep (Schrödinger, LLC). In parallel, the 3D structures of *P. falciparum* CDPK1, CDPK4, and PK6 were prepared using the Protein Preparation Wizard available on Maestro workspace (Schrödinger LLC) as follows: bond orders and formal charges were adjusted; hydrogen atoms were added to the proteins; and protonation state of polar amino acids were predicted by PROPKA (Schrödinger, LLC) (Søndergaard et al., 2011) at neutral pHs. Before docking studies, grids were established to each protein ruled by a box space of 10 × 10 × 10 Å^3^, and fixing the box on the geometrical center of ATP-binding site using the receptor grid generation panel of the Glide (Schrödinger, LLC) (Friesner et al., 2004). Finally, molecular docking calculations were carried out using Glide Extra Precision (XP) mode and constraints into hinge region. The docking poses of each virtual hit were submitted to Prime (Schrödinger, LLC) for rescoring using the Molecular Mechanics/Generalized Born Surface Area (MMGBSA) approach with default conditions.

### Experimental

#### *Plasmodium* Culture

Parasite cultures (3D7 and Dd2 strains) were maintained in O^+^ human erythrocytes in RPMI 1640 medium supplemented with 0.05 mg/mL gentamycin, 38.4 mM HEPES, 0.2% sodium bicarbonate, and 10% O^+^ human serum as described before (Trager and Jensen, 1976). To achieve a synchronic culture in the ring stage, two consecutive treatments at 48 h intervals with a 5% solution of D-sorbitol were done (Lambros and Vanderberg, 1979).

#### Determination of *Plasmodium* Growth Inhibition by SYBR Green I

Synchronized ring-stage (> 90%) infected erythrocytes were dispensed in duplicate into 96-well plates (0.5% parasitemia, 1% hematocrit) and incubated in dose response format with test compounds for 72 h. Chloroquine was used as an antimalarial control and uninfected erythrocytes as negative control. Then, *in vitro* susceptibility of parasite to tested drug was measured by SYBR Green (Hartwig et al., 2013). Following incubation, the plates were frozen and thawed, and 100 μL of the culture were transferred to a new black 96-well plate containing 100 μL of lysis buffer (20 mM Tris, 5 mM EDTA, 0.008% wt/vol saponin, 0.08% vol/vol Triton X-100, and 0.4 μL/mL of SYBR Green). After 1 h, the fluorescence was measured at 490 nm excitation and 540 nm emission (CLARIOstar, Labtech BMG). The results were compared with control cultures with no drugs. The EC_50_ was calculated by plotting the Log doses vs. Inhibition (expressed as a percentage relative to the control) in Prism 6 (GraphPad Software Inc.). Each test was performed at least three independent experiments.

#### Cytotoxicity Assay

The cytotoxicity was evaluated using two different lineages of mammalian cells: fibroblast-like cell lines derived from monkey kidney tissue (COS7 cells) and human hepatoma cell line (HEPG2). The cells were grown in 75 cm^2^ flasks containing DMEM medium supplemented with 10% fetal bovine serum and 0.05 mg/mL gentamicin under a 5% CO_2_ atmosphere at 37°C. After harvest of cells, 100 μL aliquots were distributed in 96-well plates (1 x 10^4^ cells per well) and incubated until adhesion (~12 h). The compounds at various concentrations (100−0.048 μM) were placed in the wells in duplicate and incubated for 72 h. The cell viability analysis were done by the MMT reduction method (3-[4,5-dimethyl-thiazol-2-yl]-2,5-diphenyltetrazolium chloride), after the incubation period (Mosmann, 1983). The optical density was determined at 570 nm (CLARIOstar, Labtech BMG) and the 50% cytotoxicity concentrations (CC_50_) were expressed as the percent viability relative to the control (untreated cells). The selectivity index of the compounds was determined through the ratio of the CC_50_ of both cytotoxicity results (COS7 and HEPG2 cells) and EC_50_ 3D7, separately. Experiments were performed at least three times.

#### Inhibition of *P. berghei* Sexual Stage Progression

Balb/c mice were infected intraperitoneally with the *P. berghei* Ookluc line (Calit et al., 2018). Four to five days after infection, the infected blood was collected by cardiac puncture and 4 μL were seeded to a volume of 80 μL of ookinete medium (Blagborough et al., 2013), at 21°C, containing 10 μM of compounds or DMSO vehicle control. After 24h incubation at 21°C, the nLuc substrate (Nano-Glo, Promega) was added to each well 1:1 (v:v) and incubated for 5 min at 37°C. The luciferase activity was measured using a plate luminometer SpectraMax i3; Molecular Devices and the % of conversion inhibition were calculated relative to the luciferase activity in the control assays. This assay was approved by the Ethics Committee (protocol number 132/2014-CEUA) of the Institute of Biomedical Sciences—University of São Paulo.

## Results and Discussion

### Shape-Based Models

Shape-based models were built to distinguish active vs. inactive compounds for *P. falciparum* CDPK1, CDPK4, and PK6. Initially, the chemical structures of most potent inhibitors of each protein kinase were used as queries to develop shape-based models (Table S1). Molecular conformations of queries were selected according to energy minimization. Subsequently, the ability of the models to differentiate between the active and inactive compounds was inspected. Details of model performance are shown in Table 1. As observed, all models led to AUC values ranging between 0.69 and 0.95.

**Table 1:** Validation of shape-based models using different queries.

**Kinase**	**Model**	**AUC**	**TOP 1%**		**TOP 5%**		**TOP 10%**	
			**EF**	**BEDROC**	**EF**	**BEDROC**	**EF**	**BEDROC**
CDPK1	I^*^	0.81	22.10	0.63	10.06	0.50	5.41	0.52
	II	0.83	20.99	0.58	7.40	0.41	4.74	0.45
	III	0.69	0.55	0.02	2.20	0.09	2.21	0.16
CDPK4	IV^*^	0.77	3.64	0.13	3.64	0.17	3.27	0.24
	V	0.72	3.64	0.13	3.15	0.15	3.27	0.23
	VI	0.74	3.64	0.13	3.39	0.15	3.27	0.23
PK6	VII^*^	0.95	26.15	0.77	13.64	0.68	8.46	0.74
	VIII	0.94	26.15	0.75	13.54	0.66	8.62	0.72
	IX	0.93	24.62	0.65	13.54	0.64	8.31	0.72

*AUC, area under the ROC curve; EF, Enrichmenent Factor; BEDROC, Boltzmann-Enhanced Discrimination of ROC*.

* *Selected model*.

Model I showed the best statistical performance for CDPK1, with EF values of 22.10, 10.06, and 5.41; and BEDROC values of 0.63, 0.50, and 0.52 at the top 1, 5, and 10% of the ranked database, respectively. The model IV showed the best statistical performance for CDPK4, with EF values of 3.64, 3.64, and 3.27; and BEDROC values of 0.13, 0.17, and 0.24 at the top 1, 5, and 10% of the ranked database, respectively. Finally, the model VII showed the best statistical performance for PK6, with EF values of 26.15, 13.64, and 8.46; and BEDROC values of 0.77, 0.68, and 0.74 at the top 1, 5, and 10% of the ranked database, respectively. These results indicated that our shape-based models were statistically robust and therefore would be considered for a subsequent virtual screening study.

### ML Models

ML models were built to distinguish active vs. inactive compounds for *P. falciparum* sensitive (3D7) and resistant strains (W2). According to the statistical results of the 5-fold external cross-validation procedure, the combination of Avalon, MACCS, Morgan, FeatMorgan, AtomPair fingerprints with Random Forest algorithm led to predictive ML models, with CCR values ranging between 0.70 and 0.76. Table 2 shows the detailed performances of the binary ML models.

**Table 2:** Summarized statistical characteristics of ML models accessed by 5-fold cross validation.

**Model**	**CCR**	**SE**	**SP**	**PPV**	**NPV**	**Coverage**
***P. falciparum*** **3D7 strain**						
Avalon	0.75	0.72	0.78	0.76	0.73	0.99
MACCS	0.74	0.73	0.75	0.74	0.73	1.00
Morgan	0.75	0.69	0.80	0.78	0.72	0.99
FeatMorgan	0.75	0.71	0.79	0.77	0.73	0.99
AtomPair	0.73	0.70	0.77	0.75	0.72	0.99
Consensus	0.76	0.71	0.80	0.78	0.73	1.00
Consensus rigor	0.76	0.72	0.80	0.78	0.74	0.98
***P. falciparum*** **W2 strain**						
Avalon	0.71	0.67	0.75	0.73	0.70	0.99
MACCS	0.70	0.70	0.70	0.70	0.70	1.00
Morgan	0.71	0.66	0.76	0.73	0.69	0.99
FeatMorgan	0.71	0.68	0.74	0.72	0.70	0.99
AtomPair	0.70	0.69	0.71	0.70	0.69	0.99
Consensus	0.72	0.68	0.76	0.74	0.70	1.00
Consensus rigor	0.72	0.68	0.76	0.74	0.70	0.98

*CCR, Correct Classification Rate; SE, Sensitivity; SP, Specificity; PPV, Positive Predictive Value; NPV, Negative Predictive Value*.

The model built using Avalon (CCR = 0.75, SE = 0.72, SP = 0.78, PPV = 0.76, and NPV = 0.73) and Morgan (CCR = 0.75, SE = 0.69, SP = 0.80, PPV = 0.78, and NPV = 0.72) demonstrated the best performances among all other models developed for *P. falciparum* 3D7 strain. On the other hand, the best model developed for prediction activity against W2 strain was built using Avalon (CCR = 0.71, SE = 0.67, SP = 0.75, PPV = 0.73, NPV = 0.70), Morgan (CCR = 0.71, SE = 0.66, SP = 0.76, PPV = 0.73, NPV = 0.69), and FeatMorgan (CCR = 0.71, SE = 0.68, SP = 0.74, PPV = 0.72, NPV = 0.70). Subsequently, 10 rounds of Y-randomization were performed for each data set (Table S2). The results from this analysis (CCR, SE, SP values around 0.50) indicate that predictivity of our models was not due to chance correlation.

### Virtual Screening

**Figure 2:**
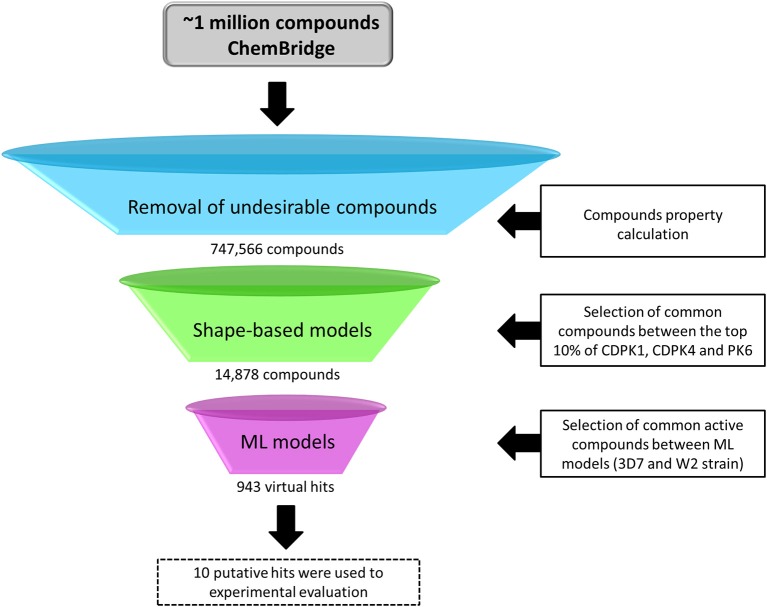
General workflow of the virtual screening campaign.

The virtual screening (VS) was carried out following the workflow presented in Figure 2. Initially, 1,091,088 compounds available on ChemBridge database were downloaded. Then, 747,566 molecules with probable oral bioavailability were prioritized using a drug-likeness filter. Then, conformers and AM1-BCC charges were generated for each molecule. The best shape-based models were used to prioritize potential *P. falciparum* multi-kinase inhibitors. Subsequently, the 14,878 common structures in top 10% scored list by shape-based filters were submitted to developed ML models for prediction of antiplasmodial activity against sensitive and resistant strains. In addition, the AD was determined in order to set “reliable” and “unreliable” predictions (Netzeva et al., 2005; Gadaleta et al., 2016). The predictions were considered reliable when the virtual hits are within the chemical space of compounds used to train ML models. At the end of this process, ten putative hits were selected for biological evaluation.

### Experimental

**Figure 3:**
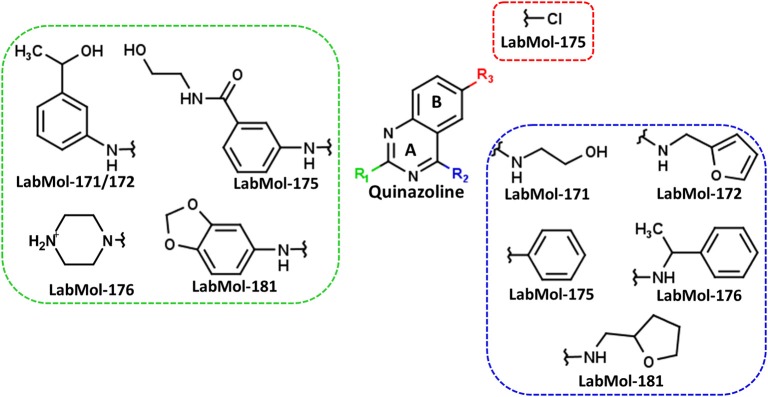
Common quinazoline scaffold between the discovered hits. The dashed green, blue and red lines indicate the substituents in R1, R2, and R3 positions, respectively.

The ten virtual hits were evaluated *in vitro* against asexual blood stages of *P. falciparum* sensitive (3D7), and multi-drug-resistant (Dd2) strains. The EC_50_ for each compound (Table 3) indicate that three compounds (LabMol-171, LabMol-172 and LabMol-181) were potent at inhibiting the parasite growth showing activities in nanomolar range against both 3D7 and Dd2 strains. These results corroborate with go/no-go criteria established for the progression of *P. falciparum* kinase inhibitors and antiplasmodial hits in VS, since the three compounds showed EC_50_ <1 μM. The compound LabMol-181 (EC_50_ = 0.39 and 0.40 μM for 3D7 and Dd2, respectively) showed the most potent activity, when compared with reference drugs, chloroquine (EC_50_ = 0.02 and 0.15 μM for 3D7 and Dd2, respectively). Moreover, the three most active compounds (LabMol-171, LabMol-172 and LabMol-181) also have a common scaffold (quinazoline), varying groups at the R1 and R2 positions (Figure 3). In contrast, LabMol-175 (EC_50_ 3D7 > 5 μM) and LabMol-176 (EC_50_ 3D7 = 1.15 μM), which also display quinazoline scaffold, shown reduced inhibition activity against chloroquine-sensitive strain. This fact can be explained mainly by the presence of hydrophobic substituents in position R2 for both compounds, and an electron withdrawing group (Cl) attached to ring B in LabMol-175.

**Table 3:** *In vitro* evaluation of selected virtual hits against asexual blood stage of *P. falciparum* 3D7 e Dd2 strains, cytotoxicity on mammalian cells (COS7, HEPG2), selectivity index and inhibition of ookinete formation of *P. berghei*.

**Structure**	**EC_**50**_ 3D7 (μM)**	**EC_**50**_ Dd2 (μM)**	**CC_**50**_ COS7 (μM)**	**CC_**50**_ HEPG2 (μM)**	SI*	SI**	**% ookinete conversion inhibition (10 μM)**
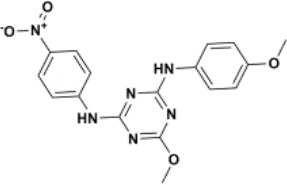	8.77 ± 2.20	3.40 ± 1.68	44.60 ± 3.73	19.95 ± 2.26	5.09	2.27	8.58 ± 7.62
LabMol-169						
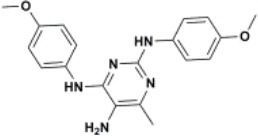	1.14 ± 0.20	2.02 ± 0.36	7.59 ± 4.09	5.43 ± 2.39	6.66	4.76	11.24 ± 19.47
LabMol-170						
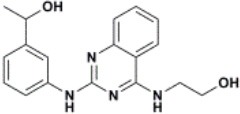	0.35 ± 0.08	0.70 ± 0.41	48.39 ± 14.04	19.31 ± 3.69	138.26	55.17	70.02 ± 22.16
LabMol-171						
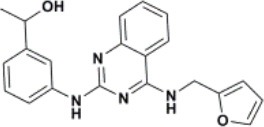	0.43 ± 0.08	0.48 ± 0.17	12.11 ± 1.44	10.21 ± 5.53	28.16	23.74	8.59 ± 13.01
LabMol-172						
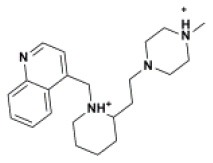	>5	–	–	–	–	–	0.00 ± 0.00
LabMol-173						
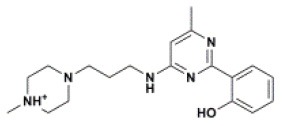	>5	–	–	–	–	–	21.41 ± 36.67
LabMol-174						
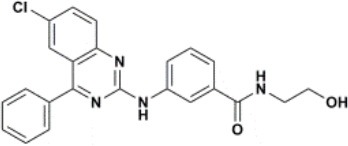	>5	–	–	–	–	–	12.87 ± 22.29
LabMol-175						
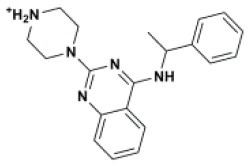	1.15 ± 0.26	1.71 ± 0.40	34.88 ± 4.06	5.63 ± 1.51	30.33	4.90	20.51 ± 19.45
LabMol-176						
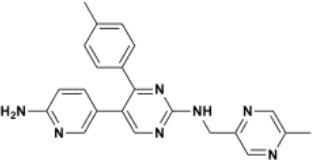	>5	–	–	–	–	–	8.76 ± 15.18
LabMol-177						
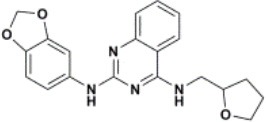	0.39 ± 0.06	0.40 ± 0.10	18.29 ± 1.92	6.13 ± 2.05	46.90	15.72	51.81 ± 23.16
LabMol-181						
Chloroquine	0.02 ± 0.01	0.15 ± 0.04	–	–	–	–	–

*EC_50_ 3D7, half maximal effective concentration in 3D7 strain; EC_50_ Dd2, half maximal effective concentration in Dd2 strain; CC_50_ COS7, half maximal cytotoxic concentration on COS7 cell; CC_50_ HEPG2, half maximal cytotoxic concentration on HEPG2 cell*;

SI^*^, selectivity index calculated between CC_50_ COS7 and EC_50_ 3D7 strain;

SI^**^, selectivity index calculated between CC_50_ HEPG2 and EC_50_ 3D7 strain. The data are expressed as mean ± SD of three independent assays.

The selected compounds were also evaluated for their cytotoxicity against fibroblast-like cell lines derived from monkey kidney (COS-7 cells) and human hepatocytes (HEPG2 cells). With respect to selectivity, LabMol-171 and LabMol-172 showed the most promising results, since they showed selectivity index (SI) ranging between 23.74 and 138.26 (Table 3). It is worth noting that no compound showed cross-resistance with multi-drug resistant strain (Dd2 EC_50_/3D7 EC_50_ ≤ 2 for all compounds), thus suggesting a different mechanism of action from clinically established antimalarial drugs.

Previous reports have demonstrated that CDPK1 e CDPK4 have critical rule for parasite gametogenesis, displaying a potential target for development of transmission-blocking drugs (Billker et al., 2004; Bansal et al., 2018). Since CDPK1 and CDPK4 compose the present multi-target approach, we decided to evaluate the potential of these compounds in inhibiting the formation of ookinetes *in vitro*, using a recently described *in vitro* luciferase assay (Calit et al., 2018). LabMol-171 and LabMol-181, promising selected compounds in terms of selectivity and inhibition growing of asexual blood stages, also showed considerable inhibition at 10 μM (70.02 and 51.81%, respectively) during ookinete formation in comparison to control. These results demonstrate that these compounds are active against multiple parasite stages, comprising human treatment and transmission blocking to mosquitoes.

### Rationalizing Anti-plasmodial Activity

Understanding the interaction pattern between the ligand and the protein target is essential for designing more potent and selective analogs. Here, molecular docking studies allowed us to rationalize the interaction the most potent hit with its associated protein targets.

As a crystal structure for docking execution was available only for *Pf* CDPK4 (PDB ID: 4QOX), the 3D structures of *Pf* CDPK1 and *Pf* PK6 were obtained by homology modeling process. The modeled and refined proteins were validated using MolProbity webserver (Table S3). This webserver encompass the metric clashscore (number of serious clashes per 1,000 atoms), which analyses steric overlap ≥0.4Å between non-bonded atoms that bring energy penalty; poor/favored rotamers, which evaluate the sidechain geometry conformation; outlier/favored Ramachandran, which evaluate protein backbone conformation by phi and psi backbone dihedrals; Molprobity score, which is represented by a number resulting from the combination of the clashscore, percentage of Ramanchandran not favored and percentage of bad side-chain rotamers, which reflects on a crystallographic resolution value; among others (Chen et al., 2010). After our investigation, we could conclude that clashscore and Molprobity score were within the desirable values, and 96.70–99.30% of the rotamers were in a favored state. Analyzes made for the values of Ramachandran pointed out that 97.25–98.30% of residues are within the favored region against 0.21–0.34% are classified as outliers. Thus, the overall stereochemistry and atoms conformation analysis displayed good quality of modeled kinases, approving them to use in docking studies.

**Figure 4:**
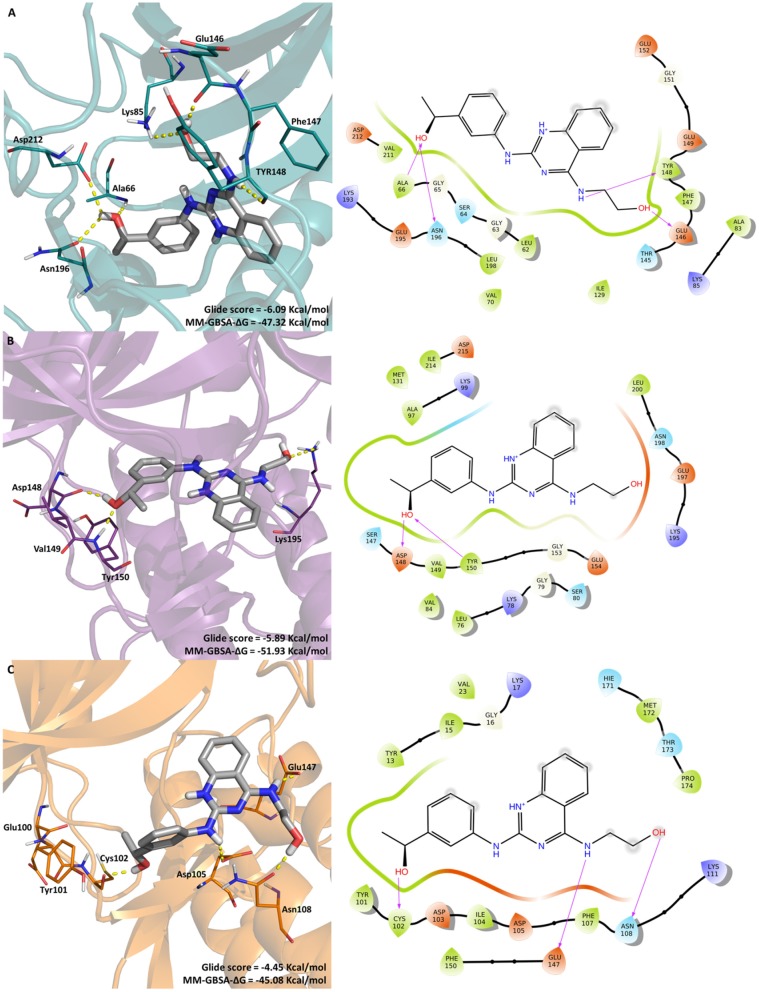
Molecular interactions of LabMol-171 with **(A)** CDPK1(cyan), **(B)** CDPK4 (purple), and **(C)** PK6 (orange) obtained by docking. In 3D representation (left), hydrogen bonds are presented as yellow dashed lines, and the color code of oxygen, nitrogen and hydrogen atoms are red, blue, and white, respectively. The carbon atoms of LabMol-171 colored as gray. In 2D interaction diagrams (right) hydrogen bond are presented as magenta arrows.

The most promising compound, LabMol-171 (EC_50_ = 0.35 μM against 3D7 and SI = 138.26 on COS7 cell) was docked into the three protein kinases (*Pf* CDPK1, *Pf* CDPK4, and *Pf* PK6) to shed some light into the interaction pattern between the ligand and the proteins. A MM-GBSA calculation was performed in order to calculate the free energy of binding. Figure 4 displays the interaction between the protein kinases and LabMol-171, the most promising experimental hit, Glide score and MMGBSA-ΔG values.

As we can see on Figure 4, the best free energy between LabMol-171 and protein kinases was obtained for calcium-dependent kinases. LabMol-171 could interact with CDPK4 (MM-GBSA-ΔG = −51.93 Kcal/mol) by hydrogen bonds at hinge region (Asp148, Tyr150) and the catalytic loop (Lys195). In relation to CDPK1 (MM-GBSA-ΔG = −47.32 Kcal/mol), hydrogen bonds were established with Lys85 and with residues belonging to the hinge region (Glu146, Tyr148), DFG motif (Asp212), catalytic loop (Asn196), and G-loop (Ala66). Aher and Roy (Aher and Roy, 2017) have showed the importance of some residues of CDPK1, including Val211, Tyr148, and Phe147 for *Pf* CDPK1 inhibitory activity.

For the docking results with PK6, a Cyclin-Dependent Kinase, LabMol-171 presented a lower Glide score (−4.45 Kcal/mol), showing high affinity with good values for free energy of binding (MM-GBSA-ΔG = −45.08 Kcal/mol). This kinase interacts with ligand in the hinge (Cys102) and catalytic loop regions (Glu147). Besides that, LabMol-171 was able to interact with Asp 105 and Asn108 of PK6.

Through our docking analysis, we could indicate that LabMol-171 could be a potential multi-kinase inhibitor, being able to interact mainly with hinge and catalytic loop region of these protein kinases. Besides that, previous studies have showed quinazoline scaffold inhibiting other molecular targets, as dihydrofolate reductase (Patel et al., 2019a,b) and prolyl-tRNA synthetase (Jain et al., 2017), besides kinases. So, prospective experimental target-fishing assays must be performed to understand the mechanism of action of quinazoline compounds in *Plasmodium*.

## Conclusion

In this work, we developed robust and predictive shape-based and machine learning models, able to prioritize 10 promising hits as antimalarial candidates. Three compounds, LabMol-171, LabMol-172 and LabMol-181, reached activity in nanomolar concentration against *P. falciparum* strains, besides low cytotoxicity on mammalian cells. Moreover, these compounds did not show cross resistance with multi-drug resistant strain, suggesting a different mechanism of action. Besides that, LabMol-171 and LabMol-181 also showed considerable inhibition of ookinete formation in *P. berghei* standing out as powerful transmission blockers. Furthermore, a docking study shed some light into LabMol-171 interactions with CDPK1, CDPK4, and PK6 and suggests that this could be a potential MKI, being able to bind with hinge and catalytic loop regions of proposed kinases. In future studies, we aim to perform enzymatic assays against CDPK1, CDPK4 and PK6, and hit-to-lead optimization studies in order to reach new MKI antimalarial drugs, with transmission blocking activity.

## Data Availability Statement

All datasets generated for this study are included in the article/Supplementary Material.

## Ethics Statement

The animal study was reviewed and approved by Ethics Committee (protocol number 132/2014-CEUA) of the Institute of Biomedical Sciences—University of São Paulo.

## Author Contributions

Each author has contributed significantly to this work. ML contributed in the design, performing the computational experiments, and writing the paper. ML, BN, and CA conceived and designed the experiments. ML, AS, and BS performed the computational experiments. GC, KT, LF, TT, JC, DB, and FC performed the experimental assays. ML, AS, BS, GC, KT, LF, TT, JC, and BN analyzed the data. ML, BN, GC, and CA wrote the paper. All authors read, edited, and approved the final manuscript.

### Conflict of Interest

The authors declare that the research was conducted in the absence of any commercial or financial relationships that could be construed as a potential conflict of interest.

## Abbreviations

MKI: multi-kinase inhibitors
CDPK1: calcium-dependent protein kinases 1
CDPK4: calcium-dependent protein kinases 4
PK6: protein kinase 6
ML: machine learning
ROC: receiver operating characteristic curve
AUC, area under the ROC curve, BEDROC: Boltzmann-enhanced discrimination of ROC
EF: enrichment factor
SE: sensitivity
SP: specificity
VS: virtual screening
CCR: correct classification rate
PPV: positive predictive value
NPV: negative predictive value
and SI: selectivity index.
