# Supplementary material for: Integrative Multi-Kinase Approach for the Identification of Potent Antiplasmodial Hits
Source: Front Chem. 2019 Nov 21;7:773. doi: 10.3389/fchem.2019.00773 (PMC6881481; doi:10.3389/fchem.2019.00773)
Supplement: Supplementary file 1 [file Data_Sheet_1.docx]

Supplementary Material

Integrative Multi-kinase Approach for the Identification of Potent Antiplasmodial Hits

Marilia N. N. Lima^1^, Gustavo C. Cassiano^2^, Kaira C. P. Tomaz^2^, Arthur C. Silva^1^, Bruna K. P. Sousa^1^, Leticia T. Ferreira^2^, Tatyana A. Tavella^2^, Juliana Calit^3^, Daniel Y. Bargieri^3^, Bruno J. Neves^4^, Fabio T.M. Costa^2^, Carolina Horta Andrade^1,2*^

^1^LabMol – Laboratory for Molecular Modeling and Drug Design, Faculty of Pharmacy, Federal University of Goiás, Goiânia, Brazil

^2^Laboratory of Tropical Diseases – Prof. Dr. Luiz Jacintho da Silva, Department of Genetics, Evolution, Microbiology and Immunology, Institute of Biology, UNICAMP, Campinas, Brazil

^3^Department of Parasitology, Institute of Biomedical Sciences, University of São Paulo, São Paulo, Brazil

^4^Laboratory of Cheminformatics, University Center of Anápolis/UniEVANGELICA, Anápolis, Brazil

*** Correspondence:**Carolina Horta Andrade
carolina@ufg.br

# Supplementary tables

**Table S1.** Selected inhibitors to generate the shape-based models

| **Kinase** | **Template Structure** | **Template Shape** |
| --- | --- | --- |
| CDPK1 |   IC_50_ = 0.007 µM  SID 307078537  Template I* | 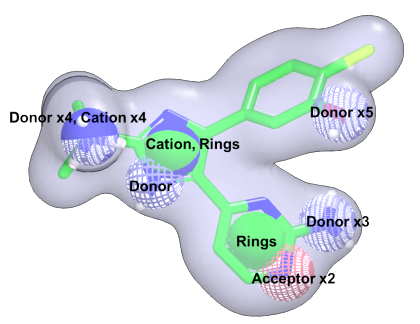 |
| CDPK1 |   IC_50_ = 0.015 µM  SID 307073749  Template II | 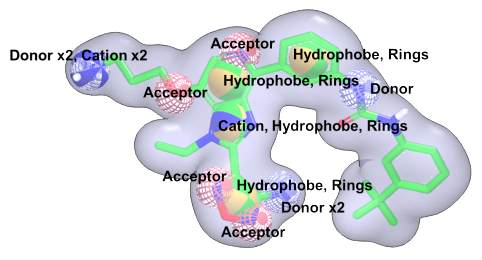 |
| CDPK1 |   IC_50_ = 0.094 µM  SID 307076176  Template III | 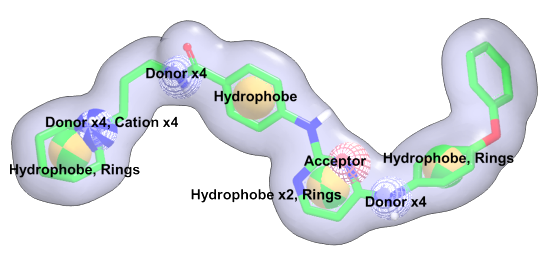 |
| CDPK4 |   IC_50_ = 0.021µM  SID 307068815  Template IV* | 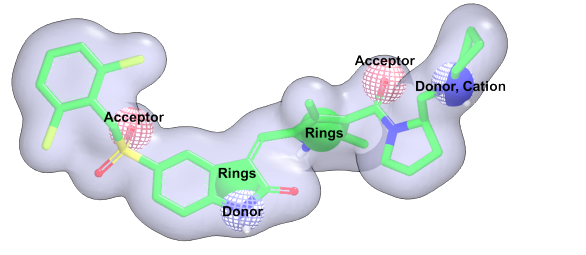 |
| CDPK4 |   IC_50_ = 0.021µM  SID 307068815  Template V | 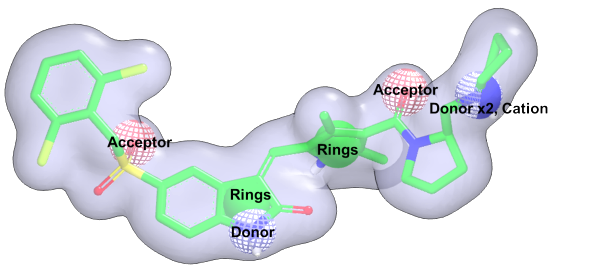 |
| CDPK4 |   IC_50_ = 0.021µM  SID 307068815  Template VI | 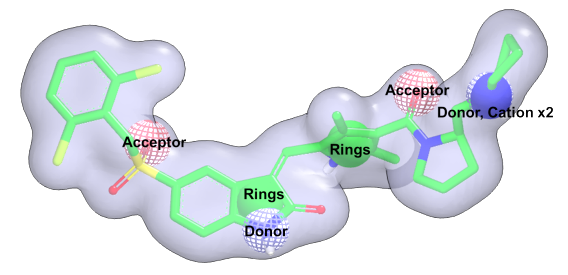 |
| PK6 |   IC_50_ = 0.042µM  SID 307071719  Template VII* | 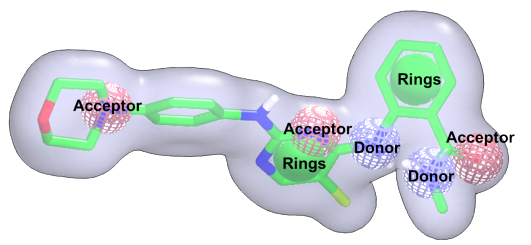 |
| PK6 |   IC_50_ = 0.042µM  SID 307071719  Template VIII | 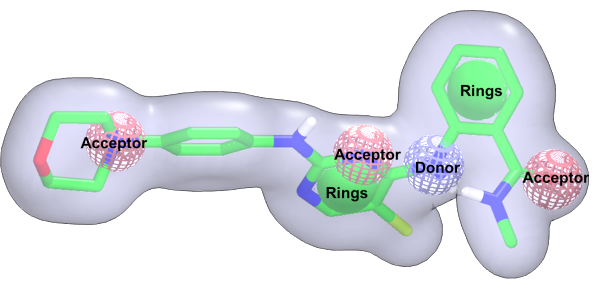 |
| PK6 |   IC_50_ = 0.042µM  SID 307071719  Template XI | 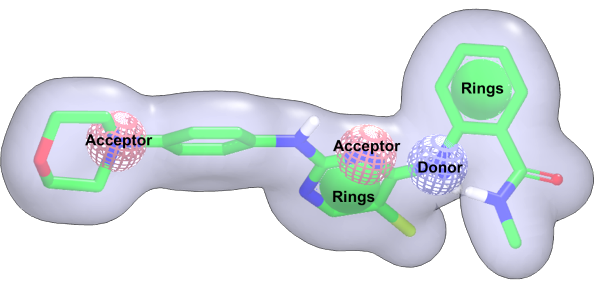 |

*Selected template.

**Table S2.** Summarized statistical characteristics of phenotypic ML models after Y-Randomization test.

| **Model** | **CCR** | **SE** | **SP** | **PPV** | **NPV** | **Coverage** |
| --- | --- | --- | --- | --- | --- | --- |
| ***P. falciparum* 3D7 strain** | | | | | | |
| Avalon | 0.51 | 0.50 | 0.51 | 0.50 | 0.52 | 1 |
| MACCS | 0.49 | 0.50 | 0.48 | 0.49 | 0.49 | 1 |
| Morgan | 0.49 | 0.46 | 0.52 | 0.49 | 0.48 | 1 |
| FeatMorgan | 0.50 | 0.50 | 0.50 | 0.50 | 0.51 | 1 |
| AtomPair | 0.51 | 0.50 | 0.53 | 0.51 | 0.51 | 1 |
| ***P. falciparum* W2 strain** | | | | | | |
| Avalon | 0.51 | 0.50 | 0.51 | 0.51 | 0.51 | 1 |
| MACCS | 0.49 | 0.51 | 0.47 | 0.49 | 0.49 | 1 |
| Morgan | 0.50 | 0.47 | 0.54 | 0.51 | 0.50 | 1 |
| FeatMorgan | 0.49 | 0.49 | 0.48 | 0.48 | 0.49 | 1 |
| AtomPair | 0.51 | 0.46 | 0.55 | 0.51 | 0.50 | 1 |

CCR: Correct Classification Rate; SE: Sensitivity; SP: Specificity; PPV: Positive Predictive Value; NPV: Negative Predictive Value; AD: Applicability Domain

**Table S3.**  Docking coordinates and validation metrics of homology modeling obtained using MolProbity webserver.

| Kinase | Template | Docking Coordinates | Validation Metrics - MolProbity | | | | | |
| --- | --- | --- | --- | --- | --- | --- | --- | --- |
|  |  |  | Clash Score | Poor rotamers | Favored rotamers | Ramachandran outliers | Ramachandran favored | MolProbity Score |
| CDPK1 | 3T3U | 19.24Å x 16.06Å x 63.58Å | 7.1 | 0.00% | 99.30% | 0.21% | 98.30% | 1.39 |
| CDPK4 | - | 31.10Å x -12.94Å x -0.14Å | - | - | - | - | - | - |
| PK6 | 3EZV | 0.70Å x 28.60Å x 9.53Å | 6.77 | 1.47% | 96.70% | 0.34% | 97.25% | 1.64 |

^*^For CDPK4 had used 4QOX crystal structure
